# Modeling and Performance Analysis of a Fault-Tolerant 3D Photonic Network-on-Chip Based on Hybrid Photonics–Plasmonics

**DOI:** 10.1155/2022/9615610

**Published:** 2022-07-19

**Authors:** Liang Zhixun, Xu Chuanpei, Bi Lvqing, Shi Yunying, Yi Yunfei, Hu Cong

**Affiliations:** ^1^School of Big Date and Computer, Hechi University, Hechi, Guangxi 546300, China; ^2^School of Electronic Engineering and Automation, Guilin University of Electronic Technology, Guilin, Guangxi 541004, China; ^3^School of Physics and Telecommunications Engineering, Yulin Normal University, Yulin 537000, China

## Abstract

The performance of electro-optic modulators and optical routers and their routing algorithms are the key factors affecting the performance of networks on optical chips. This paper improves the mesh 3-dimensional photonic network-on-chip (3D-PNoC) topology. An SPP hybrid silicon-based electro-optic modulator and an improved fault-tolerant SPP router are used to improve the performance of the network on an optical chip. SPP switching and SPP MRR are combined to form a fault-tolerant SPP router. On this basis, an improved genetic optimization routing algorithm is implemented on the improved mesh 3D PNoC topology, which completes the data exchange of the IP core of the network on the optical chip. Compared with the case of traditional optoelectronic devices, the performance of on-chip optical networks can be improved effectively. The simulation results show that upon the application of the improved genetic optimization routing algorithm to the improved mesh 3D PNoC topology, the average end-to-end delay is reduced by 32.9%, the throughput rate is increased by 28.5%, and the system power consumption is reduced by 27.6%. On the other hand, the average insertion loss and noise of optical routers are increased by 2.94 dB and 2.95 dB, respectively.

## 1. Introduction

With the explosive growth of data quantity and high-flux/high-performance computing requiring urgent, a single chip tends to integrate multiple processor cores [1–3]. There is a need for a large amount of data communication within the kernel; as is often the case, the kernel passes through the bus routes between mutual connections, but this bus connection mode needs to manage the clock synchronization [4]. When the kernel number reaches a certain amount, the clock synchronization severe delays and electromagnetic interference arise. Therefore, strongly limiting the clock frequency and multiple recycles within the kernel itself results in a large delay to communicate in this way. To expand the communication capacity, in view of on-chip system defects, a computer network introduced into the on-chip system is studied. Networks on chips have become a research hotspot of computer architecture [5–8]. In the early days, electrical interconnection was used for communication over a network. However, problems of electromagnetic interference, large time delays, and high energy consumption persist, when using electrical interconnections. Therefore, this interconnection mode cannot meet the high-capacity communication requirements of multicore processors. To solve these problems, an optical waveguide is proposed to complete the information transmission between the inner cores [9–11]. Optical waveguides have the advantages of large capacity, low delay, and low energy consumption [12]. With these advantages, issues of transmission bandwidth, crosstalk and power consumption can be effectively solved. Typical on-chip networks include mesh, torus, and octagon. These topologies have 2D and 3D structures. The 3D topology is connected by Through Silica Vias (TSVs), which expands the number of on-chip cores, reduces the chip area, and reduces the communication distance between cores. Mesh topology, as a typical optical on-chip network topology, has good connectivity and topology rules and is easy to manufacture. Key components such as network routers on optical chips are usually composed of traditional Mach-Zehnder interferomete (MZI) and Microring Resonator (MRR) switching units [13–15]. Jia et al. designed a six-port optical switch based on 12 thermally tuned silicon MZI optical switching units [15]. The ER of this switch was larger than 13.5 dB in the wavelength range from 1525 nm to 1565 nm. A data transmission rate of 32 Gbps was implemented for each optical link. Li et al. [16] proposed a low power consumption and low loss hybrid packet-circuit switched router based on microring resonator-based switching elements. However, routers composed of MZI and MRR switching units generally have large size, poor thermal stability, and low switching rate [17–19]. In recent years, the fabrication of optical devices based on the principle of surface plasmon resonance (SPR) has made it possible to fabricate devices with a wavelength of light, which not only effectively reduces the size of the device but also further reduces the power consumption and improves the working frequency. Sun et al. [20] proposed a hybrid photonic-plasmonic switch (HPPS) based on which a 5 × 5 router consists of 8 switches. Tang et al. [21] designed a 5 × 5 router for mesh topology by using HPPS. The designed router had low insertion loss and compact device size. However, some ports occupied the same switch, resulting in congestion among ports and affecting data transmission efficiency. In reference [22], we designed a high-performance full-duplex 5 × 5 router based on hybrid photonics–plasmonics and applied it to a two-dimensional mesh network. The performance analysis of the router was carried out, and the experimental results showed that its performance is superior. To apply in a 3D network, we designed three kinds of routers, namely, 3 × 3 vertical optical routers and 4 × 4- and 5 × 5-layer internal optical routers, and improved the designed router to provide fault tolerance capability. The main contributions of our paper are summarized as follows:

1. A high-performance electro-optic modulator and router are introduced into the network system on an optical chip.
2. We improve the route composition mode, reduce the load of the main router, and implement a load balancing system structure.
3. An algorithm for the congestion reduction of concurrent routers is proposed, and the delay time is reduced by the GA optimization algorithm.
4. A fault-tolerant router is proposed by combining the SPP router and SPP MRR resonator, which effectively improves system reliability.

## 2. 3D-Mesh PNoC System

### 2.1. Integrated Design

**Figure 1:**
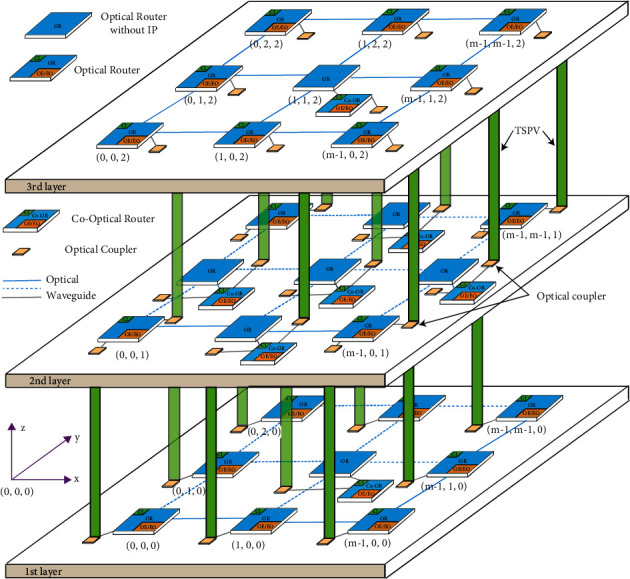
3D-PNoC with load balancing.

Many on-chip optical network topologies have been proposed by scholars, among which 2D-mesh and 3D-mesh topologies are among the most classic on-chip network topologies, which are relatively easy to implement in the manufacturing process. In this paper, we discuss the classical 3D mesh topology and refer to the design of the literature [23]. The scale of the network topology is *M* × *M* × 3, as shown in Figure 1. Address assignment starts from the coordinates in the figure. There are three layers of 3D PNOC, and each layer has a photoelectric hybrid layer for mutual mapping, the coordinates of the first layer range from (0, 0, 0) to (*m* − 1, *m* − 1, 0), the coordinates of the second layer range from (0, 0, 1) to (*m* − 1, *m* − 1, 1), and the coordinates of the third layer range from (0, 0, 0) to (*m* − 1, *m* − 1, 0). Layers are connected by TSV, and optical couplers are set. Different from the usual 3D-PNoC design, except for the edge optical router, the IP kernel is no longer mounted. Therefore, the core router with large data traffic has the function of connecting IPs only. At the same time, the corouter is responsible for completing the vertical routing task and reducing the number of ports on a single router. On the one hand, this approach balances the load balance and minimizes the number of hotspot routers. On the other hand, it reduces the complexity of a single optical router and reduces the loss and crosstalk caused by the optical waveguide cross of a single router.

### 2.2. Routing Process and Congestion Relief Method

**Figure 2:**
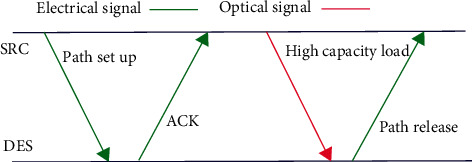
Data transmission process of the electro-optic hybrid on-chip network.

In the literature [24–26], the routing scheme shown in Figure 2 is adopted for electro-optical hybrid on-chip networks. First, the electrical layer completes optical link establishment. The process is that the source IP kernel of the electrical layer initiates the PATH setup instruction according to specific routing rules, the target IP kernel receives the PATH setup instruction, and the ACK instruction is returned along the original path. After the optical link is established, a large number of packets are transmitted through the established optical link. After data transmission, the target IP kernel returns to the path release instruction to release the optical link.

**Figure 3:**
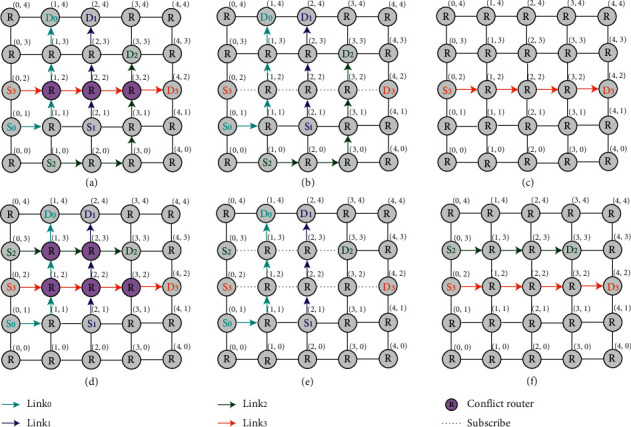
Schematic diagram of optimization of routing algorithm.

The advantages and disadvantages of the routing algorithm are evaluated by end-to-end delay, throughput, and power consumption. Under normal circumstances, communication links with conflicts are suspended to enter the waiting list and complete communication in sequence, which is improved, as shown in Figure 3. Figures 3(a) and 3(d) shows the initial states of the routing links with conflicts. By merging the communication links without conflicts, these links can be transmitted preferentially to reduce delay and power consumption and improve throughput rate. In Figures 3(b) and 3(e), links S0 -> D0–S2 -> D2 and S0 -> D0–S1 -> D1 are preferentially transmitted.

Due to the large amount of concurrent network data transmission on optical chips, we designed an improved genetic algorithm optimization algorithm to solve the above problems. In the crossover and mutation stage, we adopted the crossover and mutation operation with enlightening information orientation. The specific algorithm design is as follows.

Coding design: according to the requirements, a certain number of communication combinations of source and destination addresses are randomly generated. Each source node ID corresponds to a destination node ID, and ID is represented as a chromosome in an individual. Each individual is composed of all randomly generated source node IDS and destination node IDs. Each individual generated from the initial population must contain all randomly generated combinations of source and destination node IDs.
Fitness function design:
  (1) $$\begin{array}{c} \min:\left(f\left(p\right)\right)=\Upsilon T_{\text{delay}}+\frac{\Theta }{T_{\text{throughput}}}+\Psi E_{\text{total}},\\ ST:3\langle x\langle m-1,3\langle y\langle m-1,z=3,\\ \left(x_{sm},y_{sm},z_{sm}\right)\neq \left(x_{dm},y_{dm},z_{dm}\right),\\ \left(x_{sn},y_{sn},z_{sn}\right)\neq \left(x_{dn},y_{dn},z_{dn}\right),\\ \forall \;x,y,\;z\in \text{Random}\_\text{Add}. \end{array}$$
Select operation: with the roulette method, regarding the selected probability according to fitness size distribution, a higher fitness is more likely to be selected; therefore, there is a high likelihood of choosing the fitness of individuals at crossover and mutation operations, and smaller individual fitness values are eliminated at some probability.

**Figure 4:**
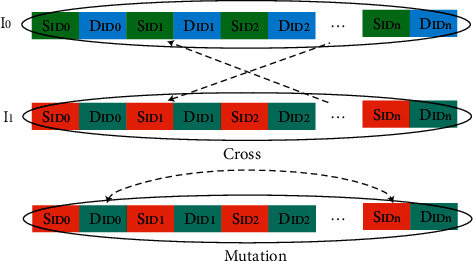
CROSS mutation operation.

As shown in Figure 4, crossover mutation operation, each individual is equivalent to a routing table. The smaller the ID number is the higher the routing priority. The link without conflict has the priority of exchanging data and iterating continuously. In the process of crossover and mutation, each individual cannot lose the randomly generated links. The chromosomes in the selected individuals are preferentially crossed according to the nonconflicting links and placed at the front of the priority routing table.

**Figure 5:**
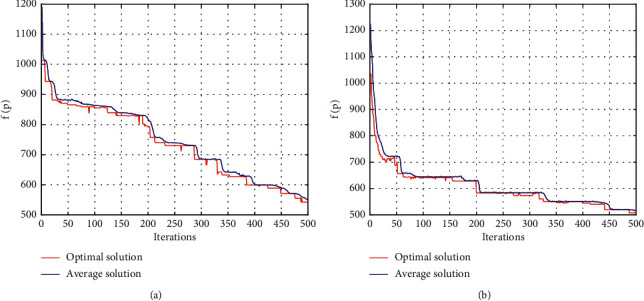
Experiment on convergence characteristics of routing algorithm using the improved genetic algorithm.

To verify the effectiveness of the improved genetic algorithm applied to the network routing algorithm on optical chips, an experimental model was established by using MATLAB. The experimental results are shown in Figure 5. Figure 5(a) shows the convergence characteristics of the ordinary *XYZ* routing algorithm, and Figure 5(b) shows the convergence characteristics of the improved genetic algorithm for the routing algorithm. According to the experimental results, the objective function *f*(*p*) of the general *XYZ* routing algorithm decreases to 600 after 450 iterations, while the objective function of the improved genetic algorithm decreases to 500 after 150 iterations. Therefore, the convergence of the improved genetic algorithm is better for the routing algorithm, which verifies that the improved genetic algorithm is effective for network routing algorithms on optical chips.

### 2.3. Design of an External Light Source and EO/OE Scheme for an Optical Network-on-Chip

**Figure 6:**
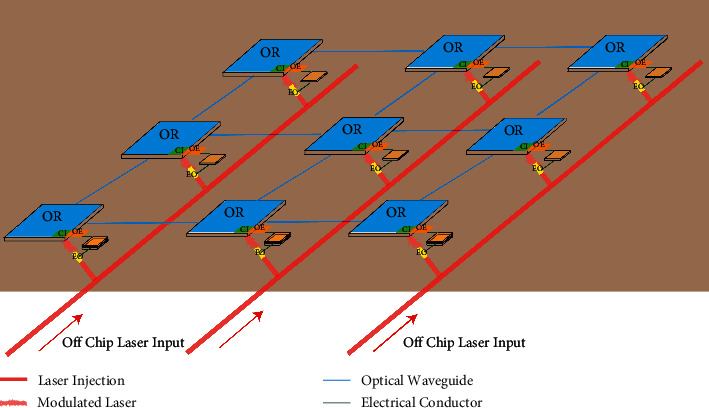
Light source outside the optical network-on-chip.

Currently, the setting scheme of light sources is divided into two types: in-film [27] and out-film [10]. The on-chip light source has low insertion loss and high integration. However, due to immature manufacturing technology, on-chip light sources are easily affected by temperature and have poor stability. Therefore, the off-chip light source is the mainstream scheme for setting the light source in the on-chip optical network. As shown in Figure 6, the light source is imported into the system from external through the optical waveguide, and the electro-optic modulator of each IP core on the chip evenly distributes the laser signal. On the one hand, when the IP kernel does not need to communicate, EO is in the OFF state and consumes only static energy. On the other hand, when the IP kernel needs to communicate, the electrical signal is converted into an optical signal through the modulator, and the signal is injected into the network. The electro-optical modulator adopts the WDM method to improve the communication capacity.

**Figure 7:**
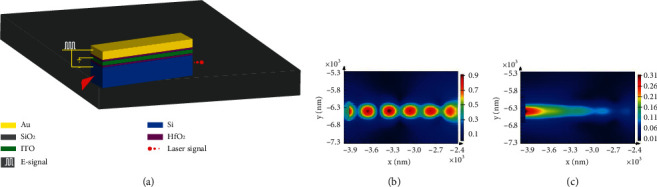
SPP electro-optic modulator.

MZI and MRR are commonly used electro-optic modulators. However, the above two types of modulators have defects such as large size, low modulation rate and susceptibility to temperature. SPP hybrid waveguide modulators are small in size, have a high modulation rate and are not easily affected by temperature [19–22], which has attracted extensive attention from scholars.  Figure 7(a) shows an SPP hybrid waveguide modulator. The dielectric constant of ITO, the activated material sandwiched with a HfO2 insulation layer, is regulated to modulate optical signals with electrical signals. Figures 7(b) and 7(c) show two states of ON and OFF in the simulation of the electro-optic modulator.

### 2.4. Full-Duplex SPP Router with Fault Tolerance

**Figure 8:**
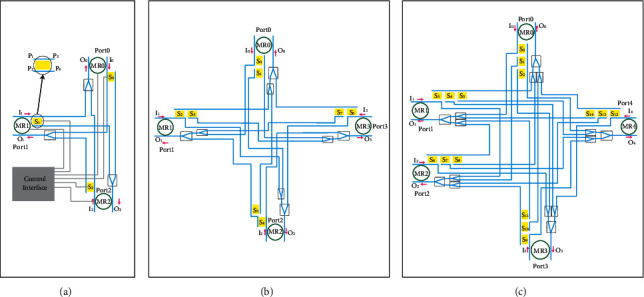
A full-duplex SPP router with fault tolerance: (a) 3 × 3 full-duplex SPP router with fault tolerance. (b) 4 × 4 full-duplex SPP router with fault tolerance. (c) 5 × 5 full-duplex SPP router with fault tolerance.

Hybrid SPP silicon waveguide modulators and switches have the advantages of small size, high modulation rate, and deeply being affected by temperature. In this paper, we design a 5 × 5 full-duplex SPP router, and simulation experiments show its superior performance. On this basis, we designed a fault-tolerant full-duplex SPP router. To meet the needs of information communication in the 3D-mesh topology, we designed three full-duplex SPP routers with different port numbers. As shown in Figures 8(a)–8(c), for 3 × 3, 4 × 4, and 5 × 5 configurations, respectively, the optical router is composed of an SPP silicon-based waveguide hybrid switch, SPP silicon-based waveguide hybrid MRR, silicon waveguide, and logic control. The SPP silicon-based waveguide hybrid switch works in cross and BAR states, while the SPP silicon-based waveguide hybrid MRR device works in DROP and BAR states. Both of them are controlled by a logical control unit to complete the routing and switching of the optical path, and the silicon-based waveguide completes the transmission of the optical signal.

**Figure 9:**
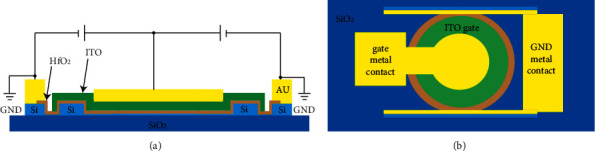
Microring resonator switch based on SPP.

Hybrid SPP silicon-based waveguide switches have been studied in the literature [20]. In this work, a hybrid SPP silicon-based waveguide MRR was studied, and its structural design is shown in Figure 9. The ordinary MRR was covered with an insulating layer of HfO2, and activated ITO was generated on the insulating layer. Au electrodes attach to the activated material layer and outer silicon-based waveguide, respectively, and voltage is applied through the two electrodes to complete the control of ITO in the activated material layer, to achieve the purpose of controlling whether the device resonates; the MRR has two states of DROP and Bar.

**Figure 10:**
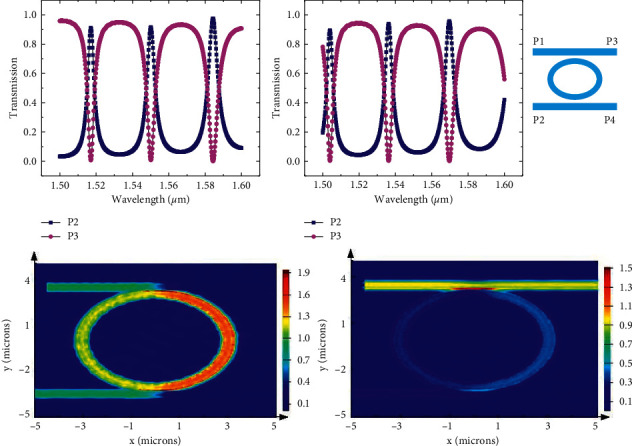
Performance simulation of a microring resonator switch based on SPP.

Then, we simulate the device and give the corresponding performance parameters. Reference [28] reported that MRR has two states of drop and bar and is completed under control voltages of 0 V and 2.35 V.  Figure 10 shows the optical power output of ports P2 and P3 in the cross and bar states under the condition of injection of an optical signal with a wavelength of 1550 nm. The transmission efficiency of port P3 in the bar state exceeds 90%, and that of port P2 in the drop state exceeds 90%. According to the numerical calculation, their insertion loss (IL) is 0.34 dB and 0.37 dB in the drop and bar states, respectively, while their crosstalk is −20 dB.

## 3. Fault Tolerance Mechanisms and Algorithms

### 3.1. Fault-Tolerant Mechanism

**Figure 11:**
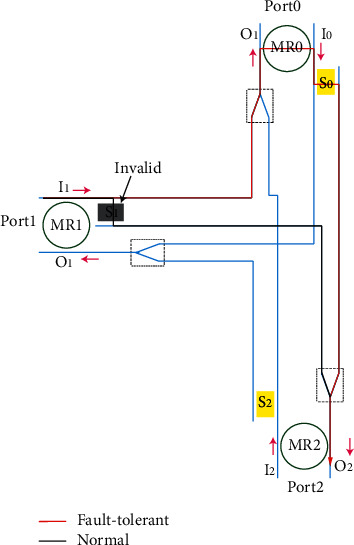
Fault-tolerant line planning.

When one of the components in the system fails, the system can still work normally after being adjusted by itself. The full-duplex 3 × 3, 4 × 4, and 5 × 5 routers with fault tolerance functions are such a system. As shown in Figure 11, the 3 × 3 full-duplex fault-tolerant router still works normally after adjustment when internal errors occur. In the figure, the router needs to realize Port1 -> Port2 link communication, but the SPP switch in the router cannot normally complete the cross state and has the function of BAR only. The system adjusts the link to Port1 -> Port0 -> Port2. MR0 resets and changes the route link. Therefore, MR0\MR1 and MR2 in the figure have no effect on the SPP switch when no error occurs and can realize redundancy and complete optical link change when error occurs.

There are three possibilities of SPP switch failure, one of which is that when a voltage is applied, the original state of the BAR changes to the cross state. The other, when no voltage is applied, should be in the cross state but change to the BAR state. The third case is that the route cannot be in either the BAR or cross state, that is, the optical signal power of the bar and CROSS output port is equal, and it is completely unavailable. Therefore, when a certain SPP switch fails, the fault tolerance mechanism has three possibilities. In this paper, the third state is not considered; only the first two states are considered and analyzed.

**Figure 12:**
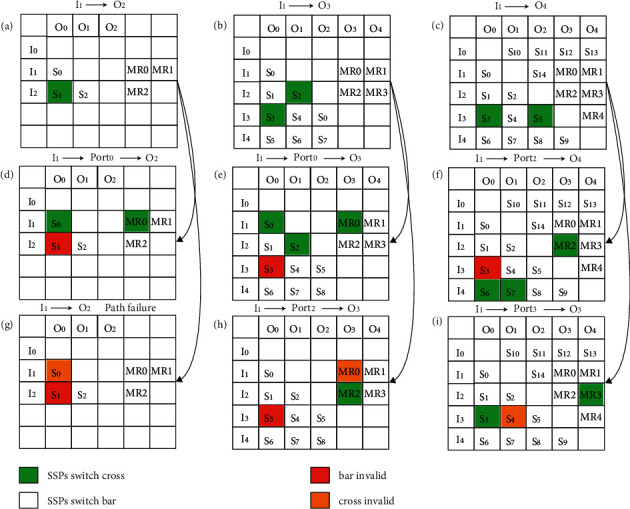
Example of a fault-tolerant routing scheme.

Figure 12 shows an example of the fault tolerance mechanism of each optical router, when SPP switch failure occurs. Figure 12(a) shows the establishment of the optical link from port 1 to port 2 in the case of the 3 × 3 route with no error on the SPP switch. In Figure 12(d), SPP switch S1 has an error; it can be in the bar state only and cannot achieve the cross state. When the detection algorithm detects this error, the error is updated to the error table. When the route needs to realize optical link communication from port 1 to port 2, it sets the link path to port 1 to port 0 to port 2 (I1 -> Port0 -> O2). In Figure 12(g), errors occur in S0 and S1, and the optical link between ports 1 and 2 fails. Figures 12(b), 12(e) and 12(h) shows the link establishment of the 4 × 4 router in the case of normal routing, one SPP switch error and two SPP switch errors, respectively. Figures 12(c), 12(f) and 12(i) show the normal route of the 5 × 5 router and the link establishment of the router in the case of one SPP switch error and in the case of two SPP switch errors, respectively. Therefore, when two or more SPP switches in a 3 × 3 router fail, the link fails. However, a 4 × 4 router can tolerate two SPP switches failing. When three or more SPP switches fail, the link fails.

### 3.2. Fault-Tolerant Routing Algorithm

Algorithm 1 is the routing algorithm designed by us for optical networks on chip with 3D-mesh topology. The symbols in the table are as follows:

- $\dot{G}\left(\dot{V}\left(G\right),\dot{W}\left(G\right)\right)$ is a set of 3 × 3 routes, where $\dot{V}\left(G\right)$ is the SPP switch and SPP MRR vectors, $\dot{V}\left(G\right)\in \left(v_{0},v_{1},v_{2},{\dot{v}}_{0},{\dot{v}}_{1},{\dot{v}}_{2}\right)$, where *v*_*n*_ is the vector of the SPP switch, ${\dot{v}}_{n}$ is the vector of the SPP MRR, and $\dot{W}\left(G\right)$ is the optical waveguide vector inside the router.
- Similarly, $\ddot{G}\left(\ddot{V}\left(G\right),\ddot{W}\left(G\right)\right)$ is a set of 4 × 4 routes, and $\dddot{G}\left(\dddot{V}\left(G\right),\dddot{W}\left(G\right)\right)$ is a set of 5 × 5 routes.
- *F*_*x*,*y*,*z*_^*n*^ records the error SPP switch and SPP MRR in the route, (*x*, *y*, *z*) is the coordinate of the error that occurred in the router, and *n* is the mark of the error SPP switch and SPP MRR in the router. In addition, new*F*_*x*,*y*,*z*_^*n*^ is the flag of the SPP switch and SPP MRR that detects errors in the router.
- Furthermore, (*n*)*rP*((*x*_*s*_, *y*_*s*_, *z*_*s*_)⟶(*x*_*d*_, *y*_*d*_, *z*_*d*_)) is the link establishment request information in the system, *n* is the number of link establishment requests in the system, (*x*_*s*_, *y*_*s*_, *z*_*s*_) is the source address required to establish communication links, and (*x*_*d*_, *y*_*d*_, *z*_*d*_) is the destination address required to establish communication links. The simulation requires (*x*_*s*_, *y*_*s*_, *z*_*s*_) ≠ (*x*_*d*_, *y*_*d*_, *z*_*d*_).
- *P*_*s*−〉*d*_ is the output of the route path after the link is established, and SPP ST (*x*, *y*, *z*) is the corresponding SPP switch and SPP MRR that can meet the status of link establishment.

Before establishing a routed link, the algorithm detects and marks the SPP switch and SPP MRR where errors occur in the router, and add new*F*_*x*,*y*,*z*_^*n*^ to *F*_*x*,*y*,*z*_^*n*^. Then, the number of requested routing links is detected. If (*n*)*rP*((*x*_*s*_, *y*_*s*_, *z*_*s*_)⟶(*x*_*d*_, *y*_*d*_, *z*_*d*_) · *n*)〉1, the improved genetic optimization algorithm is used to plan the priority of each routing link. Then, the algorithm determines whether the error list of SPP switch and SPP MRR is greater than 2. If the error list is greater than 2, an avoidance route is adopted. If the error list is greater than 0 and less than 2, the algorithm establishes a fault route link and transmits data according to the original route link. If none of the preceding conditions exist, the common routing algorithm is used to establish routing links and transmit data.

## 4. Reliability and Fault-Tolerant Performance Analysis

### 4.1. Global Reliability Analysis

Reference [23] proposed the reliability calculation method of an optical chip network system, which can be calculated according to the following formula:

(2) $$\begin{array}{c} R_{\text{ONOC}-\text{SYS}}=\prod_{i=1}^{N_{L}}R_{L}\left(i\right)\times \prod_{j=1}^{N_{\text{OR}}}{\dot{R}}_{OR}\left(j\right)\times \prod_{k=1}^{N_{\text{OR}}}{\ddot{R}}_{\text{OR}}\left(k\right)\times \prod_{l=1}^{N_{\text{OR}}}{\overset{\ldots }{R}}_{\text{OR}}\left(l\right). \end{array}$$

In the above equation, *R*_ONOC−SYS_ is the reliability of the entire system, and *R*_*L*_, ${\dot{R}}_{\text{OR}}$, ${\ddot{R}}_{\text{OR}}$, and ${\dddot{R}}_{\text{OR}}$ are the reliability of the waveguide and 3 × 3, 4 × 4, and 5 × 5 optical routers, respectively. In the on-chip networks, *R*_*L*_(*i*) is the *i*-th root guide, ${\dot{R}}_{\text{OR}}\left(j\right)$ is the *j*-th 3 × 3 router, ${\ddot{R}}_{\text{OR}}\left(k\right)$ is the *k*-th 4 × 4 router, and ${\dddot{R}}_{\text{OR}}\left(l\right)$ is the *l*-th 5 × 5 router. Among them,

(3) $$\begin{array}{c} {\dot{R}}_{\text{OR}}\text{ or }{\ddot{R}}_{\text{OR}}\text{ or }{\overset{\ldots }{R}}_{\text{OR}}=1-\sum_{m=0}^{n}\delta_{m}\left(\begin{array}{c} n\\ m \end{array}\right){\left(1-p\right)}^{m}{\left(p\right)}^{\left(n-m\right)}\times \sum_{r=0}^{s}\epsilon_{r}\left(\begin{array}{c} s\\ r \end{array}\right){\left(1-q\right)}^{r}{\left(q\right)}^{\left(s-r\right)}. \end{array}$$

In the above formula, *p* and *q* are the reliability of a single SPP switch and SPP MRR resonator, respectively, *n* and *s* record the total number of SPP switches and SPP MRR resonators in a single router, respectively, and *δ*_*m*_ and *ϵ*_*r*_ are the failure probabilities of *m* SPP switches and *r* SPP MRR resonators, respectively. In addition, *δ*_*m*_, *ϵ*_*r*_ ∈ [0,1].

For 3 × 3 optical router, *n* and *s* are 3. If there is no SPP switch or SPP MRR resonator failure or only one of them fails, where (*m* = *s* = 0 or *m* = 1 or *r* = 1), then the router can work normally; then, *δ*_0_=0, *ϵ*_0_=0 . When a communication link has an SPP switch and its corresponding fault-tolerant SPP MRR resonator fails simultaneously, where (*m* = 1 and *r* = 1), $\delta_{1}=1/\left(\begin{array}{c} 3\\ 1 \end{array}\right),\varepsilon_{0}=1/\left(\begin{array}{c} 3\\ 1 \end{array}\right)$, then the link fails and cannot communicate normally. Then, the reliability of the 3 × 3 optical router can be improved:

(4) $$\begin{array}{c} {\dot{R}}_{\text{OR}}=1-\sum_{m=0}^{3}\delta_{m}\left(\begin{array}{c} 3\\ m \end{array}\right){\left(1-p\right)}^{m}{\left(p\right)}^{3-m}\times \sum_{r=0}^{3}\epsilon_{r}\left(\begin{array}{c} 3\\ r \end{array}\right){\left(1-q\right)}^{r}{\left(q\right)}^{\left(3-r\right)}\geq 1-\left[\left(1-p\right){\left(p\right)}^{2}+\sum_{m=2}^{3}\left(\begin{array}{c} 3\\ m \end{array}\right){\left(1-p\right)}^{m}{\left(p\right)}^{3-m}\right]\times \left[\left(1-q\right){\left(q\right)}^{2}+\sum_{r=2}^{3}\left(\begin{array}{c} 3\\ r \end{array}\right){\left(1-q\right)}^{r}{\left(q\right)}^{3-r}\right]. \end{array}$$

Similarly, for a 4 × 4 optical router, *n* and *r* are 6 and 4, respectively, so the reliability of the 4 × 4 optical router is

(5) $$\begin{array}{c} {\ddot{R}}_{\text{OR}}=1-\sum_{m=0}^{6}\delta_{m}\left(\begin{array}{c} 6\\ m \end{array}\right){\left(1-p\right)}^{m}{\left(p\right)}^{6-m}\times \sum_{r=0}^{4}\epsilon_{r}\left(\begin{array}{c} 4\\ r \end{array}\right){\left(1-q\right)}^{r}{\left(q\right)}^{4-r}\geq 1-\left[\left(1-p\right){\left(p\right)}^{2}+\sum_{m=2}^{6}\left(\begin{array}{c} 6\\ m \end{array}\right){\left(1-p\right)}^{m}{\left(p\right)}^{6-m}\right]\times \left[\left(1-q\right){\left(q\right)}^{2}+\sum_{r=2}^{4}\left(\begin{array}{c} 4\\ r \end{array}\right){\left(1-q\right)}^{r}{\left(q\right)}^{4-r}\right]. \end{array}$$

Similarly, for a 5 × 5 optical router, *n* and *r* are 15 and 5, respectively, so the reliability of the 5 × 5 optical router is

(6) $$\begin{array}{c} {\overset{\ldots }{R}}_{\text{OR}}=1-\sum_{m=0}^{n}\delta_{m}\left(\begin{array}{c} n\\ m \end{array}\right){\left(1-p\right)}^{m}{\left(p\right)}^{n-m}\times \sum_{r=0}^{s}\epsilon_{r}\left(\begin{array}{c} s\\ r \end{array}\right){\left(1-q\right)}^{r}{\left(q\right)}^{s-r}\geq 1-\left[\left(1-p\right){\left(p\right)}^{2}+\sum_{m=2}^{15}\left(\begin{array}{c} 15\\ m \end{array}\right){\left(1-p\right)}^{m}{\left(p\right)}^{15-m}\right]\times \left[\left(1-q\right){\left(q\right)}^{2}+\sum_{r=2}^{5}\left(\begin{array}{c} 5\\ r \end{array}\right){\left(1-q\right)}^{r}{\left(q\right)}^{5-r}\right]. \end{array}$$

Then, the system reliability of the 3D-mesh optical network can be expressed as

(7) $$\begin{array}{c} R_{\text{ONOC}-\text{SYS}}=\prod_{j=1}^{N_{\text{OR}}}{\dot{R}}_{\text{OR}}\left(j\right)\times \prod_{k=1}^{N_{\text{OR}}}{\ddot{R}}_{\text{OR}}\left(k\right)\times \prod_{l=1}^{N_{\text{OR}}}{\overset{\ldots }{R}}_{\text{OR}}\left(l\right)\geq {\left\{1-\left[\left(1-p\right){\left(p\right)}^{2}+\sum_{m=2}^{3}\left(\begin{array}{c} 3\\ m \end{array}\right){\left(1-p\right)}^{m}{\left(p\right)}^{3-m}\right]\times \left[\left(1-q\right){\left(q\right)}^{2}+\sum_{r=2}^{3}\left(\begin{array}{c} 3\\ r \end{array}\right){\left(1-q\right)}^{r}{\left(q\right)}^{3-r}\right]\right\}}^{2\left(Μ-1\right)\times \left(Ν-1\right)}\times {\left\{1-\left[\left(1-p\right){\left(p\right)}^{2}+\sum_{m=2}^{6}\left(\begin{array}{c} 6\\ m \end{array}\right){\left(1-p\right)}^{m}{\left(p\right)}^{6-m}\right]\times \left[\left(1-q\right){\left(q\right)}^{2}+\sum_{r=2}^{4}\left(\begin{array}{c} 4\\ r \end{array}\right){\left(1-q\right)}^{r}{\left(q\right)}^{4-r}\right]\right\}}^{\left(Μ-1\right)\times \left(Ν-1\right)+12}\\ \times {\left\{1-\left[\left(1-p\right){\left(p\right)}^{2}+\sum_{m=2}^{15}\left(\begin{array}{c} 15\\ m \end{array}\right){\left(1-p\right)}^{m}{\left(p\right)}^{15-m}\right]\times \left[\left(1-q\right){\left(q\right)}^{2}+\sum_{r=2}^{5}\left(\begin{array}{c} 5\\ r \end{array}\right){\left(1-q\right)}^{r}{\left(q\right)}^{5-r}\right]\right\}}^{3\left(Μ\times Ν\right)-12}. \end{array}$$

In the above formula, the SPP switch and SPP resonator switch, the basic unit of the optical router, are made of silicon material subject to a thermal-optical effect. Therefore, we consider that the main factor of device unreliability is the thermal-optical effect [29], and the change in temperature leads to a shift in the resonant wavelength. From the literature, the dependence on temperature is 0.005 nm/°C.

**Figure 13:**
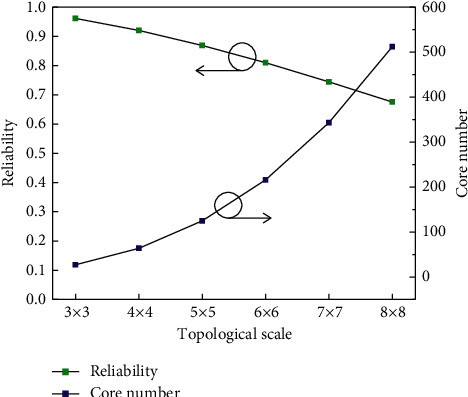
System reliability analysis curve.

System reliability consists of a reliability series of components. The more components there are, the greater the probability of failure is. To verify the reliability of the system, a simulation system is established using MATLAB according to the above theoretical derivation, and the results are drawn in Figure 13. When the size of the network on the optical chip is 3 × 3, the reliability is more than 95%; when the scale continues to increase, the reliability gradually decreases; and when the size of the network on the optical chip is 8 × 8, the reliability is 68%, still maintaining a high reliability.

### 4.2. Analysis of Overall Fault Tolerance Performance

**Figure 14:**
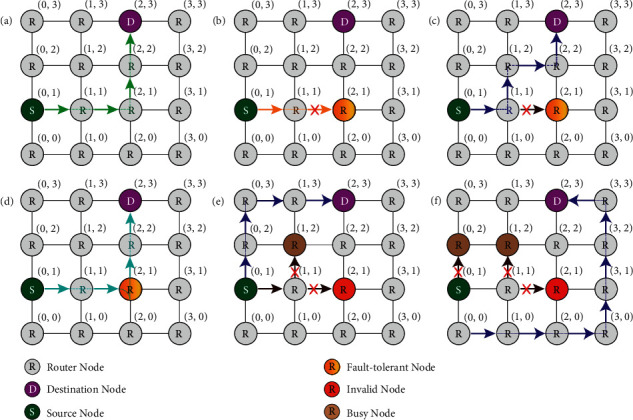
Analysis of system fault tolerance performance.

Under normal circumstances, for a common router, as shown in Figure 14, when there is no error inside the router, the route is carried out according to the usual (*x*, *y*, *z*) path. When an error occurs, “detour” is often required to avoid the router with the error. The fault-tolerant router, by means of redundant design, can still work normally when a few errors occur in the internal components of the router. As shown in Figure 12(c), when the SPP switch and SPP MRR resonator switch in the router with coordinates (2, 1) exhibits errors less than 2, the communication task can still be completed by reconfiguring the link path. Compared with the traditional router, the system does not need to increase the extra link cost, especially after the increase in the concurrency of system routes, and the system communication capacity is increased. In addition, on the basis of a fault-tolerant routing algorithm, adding a busy-aware detection routing algorithm can improve the utilization rate of idle routers to reduce route delays and improve the communication capacity of the whole system.

## 5. Overall Performance Analysis (Throughput, Delay, Energy Consumption, IL, and Noise)

This section first gives the evaluation method of throughput, delay and energy consumption performance analysis of key parameters to evaluate the on-chip network character. Then, a simulation platform for performance parameter evaluation is built in the DEV-C++ integrated development environment to simulate and analyze the above three parameters.

### 5.1. Evaluation Methodology

The throughput rate is a key parameter to measure the performance of the entire network architecture and routing policy, it is expressed by the ratio of the amount of sent data packets to the received data. Reference [30] proposed the calculation method of throughput rate as

(8) $$\begin{array}{c} {\text{T}}_{\text{throughput}}=\lambda \times \frac{PK_{\text{received}}}{PK_{\text{generated}}}. \end{array}$$

In the above formula, *λ* is the network injection rate, and its size represents the indicator of the speed of packet injection into the network. The larger the value is, the faster the packet is fired, while the smaller it is, the slower the packet is fired. *PK*_generated_ is the excitation source address of packets, and *PK*_received_ is the destination address of packets received. When *PK*_generated_〉*PK*_received_, demand gradually increases the injection rate, the survival time of packets on the network is longer. That is, the time delay is greater, and network throughput can no longer be increased but tends to a constant value, which can achieve a suitable throughput rate, with better network performance.

The average end-to-end delay is also a key parameter to measure the performance of the entire network; refer to the calculation method in the literature [30] as follows:

(9) $$\begin{array}{c} T_{\text{delay}}=\sum_{i=1}^{M}t_{i}/M, \end{array}$$

where *t*_*i*_ is the survival time of the *i*th packet on the network, that is, the length of time from triggering a packet at the destination address to receiving the packet at the source address, and *M* is the number of packets sent in a period of time. In an electro-optical hybrid network on a chip system, the factors affecting the delay include the photoelectric converter, electro-optical converter, routing path planning and system clock.

Energy consumption is an important indicator to measure the scalability and high reliability of a system. According to the data transmission behavior of the electro-optical hybrid on-chip network, the average power consumption of the system size is used to represent the system energy consumption, and the calculation method is as follows:

(10) $$\begin{array}{c} E_{\text{total}}=\frac{\sum_{i=1}^{M}\left(E_{\text{set}\_\text{up}}+E_{ACK}+E_{TR}+E_{\text{REL}}+E_{\text{Static}}\right){}^{*}T_{\text{delay}}}{3{}^{*}m{}^{*}n{}^{*}M}. \end{array}$$

In the above formula, *E*_set_up_ is the energy consumption for link establishment, *E*_ACK_ is the energy consumption for node response after receiving *E*_set_up_, *E*_TR_ is the energy consumption for data transmission, *E*_REL_ is the energy consumption for link release, and *E*_Static_ is the static power consumption of data to be sent during data transmission, 3 ^*∗*^ *m* ^*∗*^ *n* ^*∗*^ *M* is the size of the 3D-PNoC, and fJ is the unit of energy. The factors affecting power consumption are static power consumption and dynamic power consumption. Dynamic power is the power consumption generated by data transmission, while static power is the power consumption of storage data to be sent and static current consumption of electro-optical devices.

### 5.2. Parameter Simulation and Analysis

To verify the performance of the designed system, we carried out PNoC verification platform development on the Dev-C++ integrated development platform. The core code was programmed in C++ language, providing various parameter modification interfaces, and the program simulated the routing and data transmission process behavior of the electro-optical hybrid network-on-chip and could complete concurrent data request processing. This can realize the function of improving the behavior of the GA genetic routing optimization algorithm and fault-tolerant routing algorithm. The simulated on-chip optical networks range in size from 3 × 3 × 3 to 8 × 8 × 3. Following reference [22], Tables 1 and 2 show simulation parameter settings of the electrical layer and optical layer, respectively.

**Figure 15:**
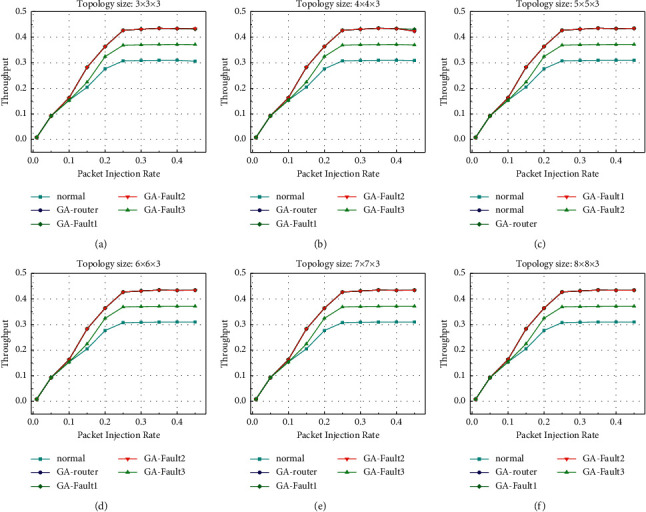
Throughput rate simulation results.

After establishing the simulation model, we first simulate the throughput of the system, and the topology scale of the simulation is 3 × three × 3 to 8 × eight × 3. Under the topology model in Figure 1, the throughput under GA-Router, GA-Fault1, GA-Fault2, and GA-Fault3 modes is simulated. In normal mode, only common *x* -> *y* -> *z* routes are used. GA refers to the adoption of an evolutionary optimization routing algorithm combined with *x* and *y* retreat and a give-way algorithm in the case of no network fault. GA-Fault1, GA-Fault2, and GA-Fault3 refer to the evolutionarily optimized routing algorithm combined with *x* and *y* retreat and a give-way algorithm when the router has one, two, and three faults, respectively. If a fault exists on a link of a 3 × 3 router, the fault tolerance mechanism works properly. If two or more faults occur, the link becomes invalid. For 4 × 4 and 5 × 5 routers with two or fewer faults on one link, the fault tolerance mechanism works normally. If more than two faults occur, the link becomes invalid. Therefore, in the GA-Fault2 and GA-Fault3 modes, two or more faults exist on one link of a router, which occur on 4 × 4 routers and 5 × 5 routers. As shown in Figure 15, when the injection rate is less than 0.1, the throughput rates of the normal, GA-Router, GA-Fault1, GA-Fault2, and GA-Fault3 modes are basically the same. During the operation of the simulation model, all kinds of data were recorded according to formula (8). After data processing was completed, the results were plotted in Figure 15. As shown in the figure, when the injection rate is less than 0.1, the throughput rates of the normal, GA-Router, GA-Fault1, GA-Fault2, and GA-Fault3 modes are basically the same. When the injection rate is greater than 0.1, the throughput increases fastest in the GA-Router, GA-Fault1, and GA-Fault2 modes, followed by the GA-Fault3 mode. The throughput increases least in normal mode. Finally, when the injection rate is greater than 0.3, the throughput of the GA-Router, GA-Fault1, and GA-Fault2 modes is constant at approximately 0.43, that of the GA-Fault3 mode is constant at approximately 0.37, and that of the normal mode is constant at approximately 0.30. The throughput of the GA-Router, GA-Fault1, and GA-Fault2 modes increases by 28.6% compared with the normal mode, and that of the F3 mode increases by 16.8% compared with the normal mode. According to the throughput performance simulation results, the routing performance of the on-chip optical network is the best after adopting the evolutionary optimization routing algorithm combined with the *X* and *Y* backoff and give-way algorithms, whether there is a router failure or not. In the case of link failure (3 faults occur in one link of routers 4 × 4 and 5 × 5), the throughput performance of the improved algorithm is still better than that of the normal mode because the *x* and *Y* backoff and give-way algorithms are adopted. When the simulation scale is from 3 × 3 × 3 to 8 × 8 × 3, the throughput performance is basically consistent, which proves that the algorithm has good consistency for on-chip networks with different topologies.

**Figure 16:**
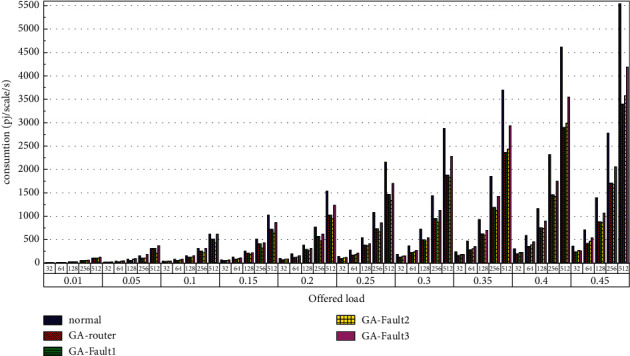
Simulation results of power consumption, when the topology scale is 3 × 3 × 3.

**Figure 17:**
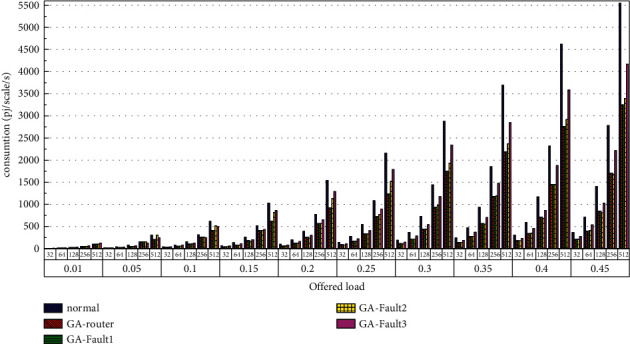
Simulation results of power consumption, when the topology scale is 4 × 4 × 3.

**Figure 18:**
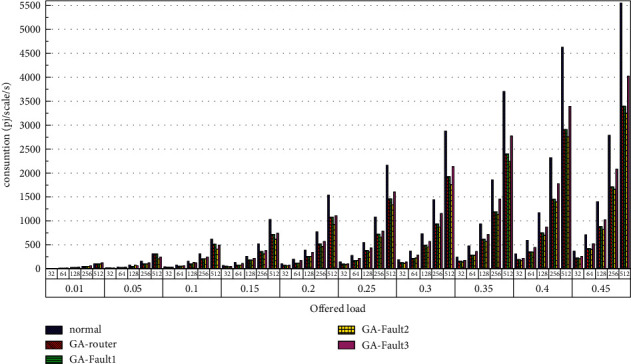
Simulation results of power consumption, when the topology scale is 5 × 5 × 3.

**Figure 19:**
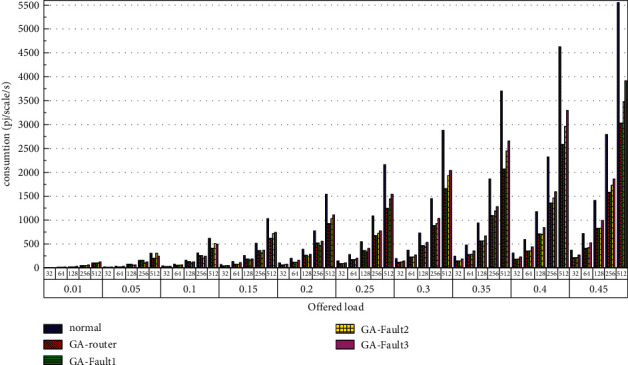
Simulation results of power consumption, when the topology scale is 6 × 6 × 3.

**Figure 20:**
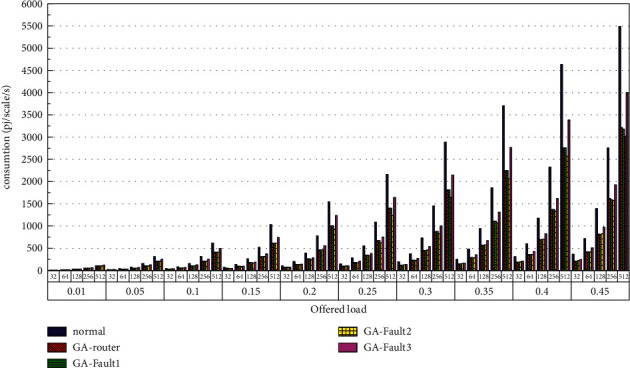
Simulation results of power consumption, when the topology scale is 7 × 7 × 3.

**Figure 21:**
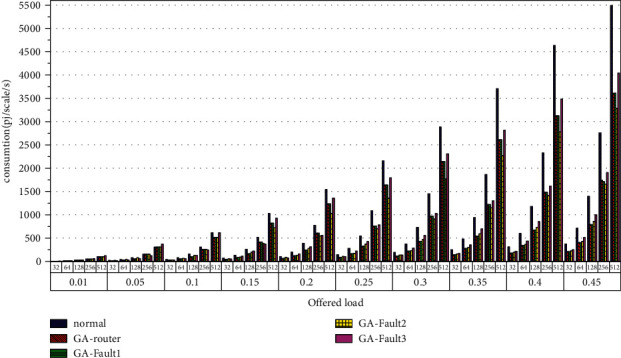
Simulation results of power consumption, when the topology scale is 8 × 8 × 3.

Then, the power consumption performance of the system is simulated. Similarly, the topology size of the network on the optical chip ranges from 3 × 3 × 3 to 8 × 8 × 3, and the packet length includes 32 bit, 64 bit, 128 bit, 256 bit and 512 bit. We set parameters according to Tables 1 and 2 and conduct experimental simulation. The experimental results are plotted in Figures 16–21. The simulation results show that the power consumption increases with a continuous increase in the injection rate. When the injection rate is fixed, the power consumption increases with increasing packet length. When the injection rate and packet length are fixed, the GA-Router, GA-Fault1, and GA-Fault2 modes have the lowest power consumption, the normal mode has the highest power consumption, and the Ga-Fault3 mode has the second-highest power consumption. Therefore, from the simulation results of the system power consumption performance, the routing performance of the on-chip optical network is the best after adopting the evolutionary optimization routing algorithm combined with the *X* and *Y* backoff and give-way algorithms, whether there is a router failure or not. In the case of link failure (three faults occur on one link of 4 × 4 and 5 × 5 routers), the improved algorithm adopts *X* and *Y* backoff and yield algorithms to reduce the waiting time of routes and static power consumption, but its power consumption performance is still better than that of the normal mode. It takes some energy to send and receive data, so the longer the packet is, the more power it consumes. After calculation, the energy consumption is reduced by 27.6% after adopting the evolutionary optimization routing algorithm combined with the *x* and *Y* retreat and yield algorithm. When the simulation scale is from 3 × 3 × 3 to 8 × 8 × 3, the power performance of the system is basically the same, and the power performance of the on-chip optical network with different topology sizes is consistent with the improved routing algorithm.

**Figure 22:**
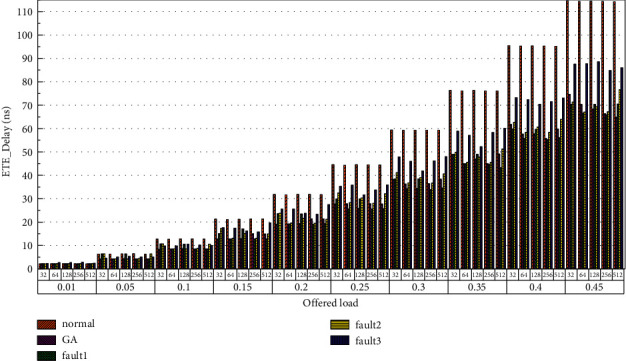
Simulation results of ETE_delays simulation results when the topology scale is 3 × 3 × 3.

**Figure 23:**
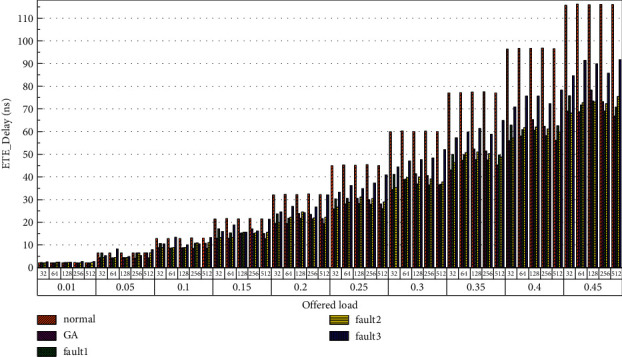
Simulation results of ETE_delays simulation results, when the topology scale is 4 × 4 × 3.

**Figure 24:**
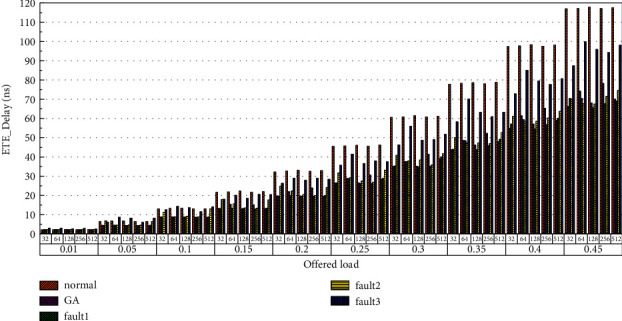
Simulation results of ETE_delays simulation results, when the topology scale is 5 × 5 × 3.

**Figure 25:**
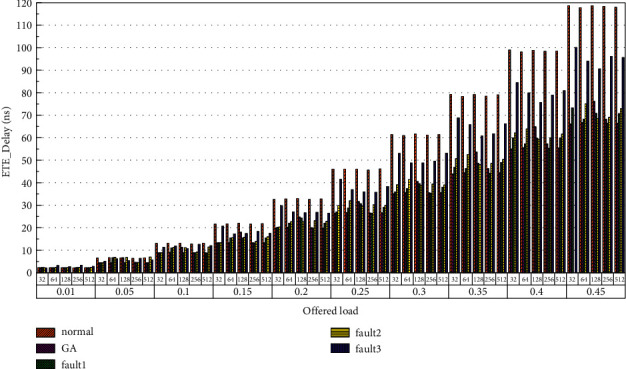
Simulation results of ETE_delays simulation results, when the topology scale is 6 × 6 × 3.

**Figure 26:**
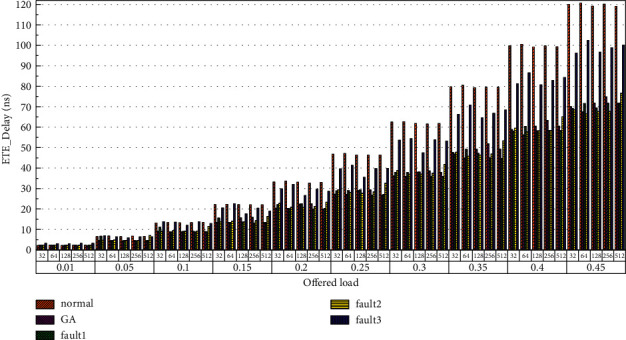
Simulation results of ETE_delays simulation results, when the topology scale is 7 × 7 × 3.

**Figure 27:**
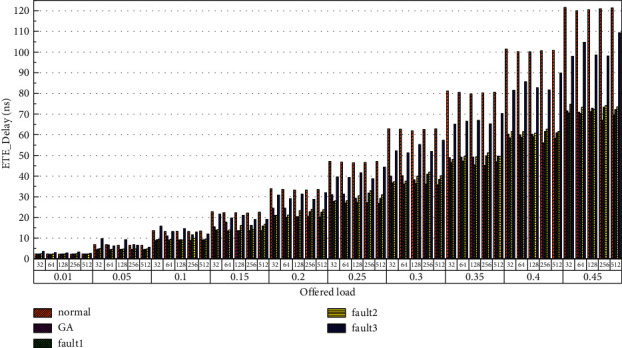
Simulation results of ETE_delays simulation results, when the topology scale is 8 × 8 × 3.

Finally, the average end-to-end delay performance of the system is simulated. The simulation model is configured according to the parameters in Tables 1 and 2, and the simulation results are plotted in Figure 22–27. According to the experimental simulation results, when the injection rate increases gradually, the average end-to-end delay increases rapidly. When the injection rate increases, the number of communication links with conflicts increases, which increases the waiting time and leads to a rapid increase in the end-to-end delay. The optical transmission rate is as high as 50 Gbps, which is much shorter than the time required for link establishment and release. Therefore, when the packet length becomes longer, the delay does not increase. This aspect also shows the application of optical transmission on the chip, which is used for large-capacity data communication, and highlights its superior performance. When the injection rate remains unchanged, the GA-Router, GA-Fault1, and GA-Fault2 modes have the lowest latency, and the normal mode has the highest power consumption, followed by GA-Fault3. Therefore, from the simulation results of system power consumption and delay, the routing performance of the on-chip optical network is optimal whether there is a router failure after adopting the evolutionary optimization routing algorithm combined with the *x* and *y* backoff and give-way algorithms. In the case of link failure (3 faults occur on one link of routers 4 × 4 and 5 × 5), the power consumption performance of the improved algorithm is still better than that of the normal mode because the *x* and *Y* backoff and yield algorithms are adopted to reduce the waiting time and delay of routes. After calculation, the time delay is reduced by 32.9% after adopting the evolutionary optimization routing algorithm combined with the *X* and *Y* backoff and give-way algorithms. When the simulation scale is from 3 × 3 × 3 to 8 × 8 × 3, the delay performance of the system is basically consistent, and the improved routing algorithm has good consistency of the delay performance of the on-chip optical network with different topologies.

## 6. Optical Performance Analysis (Crosstalk, IL, Etc.)

Optical crosstalk and IL are important parameters of optical communication links in optical routers, which affect the communication quality and scalability of networks on optical chips. Therefore, this section studies the optical performance of fault-tolerant routers based on these two parameters. According to reference [22], optical crosstalk is formulated as

(11) $$\begin{array}{c} CN_{T}=10\text{log}\left(\sum CN_{1}+\sum CN_{2}\right). \end{array}$$

In the above formula,

(12) $$\begin{array}{c} CN_{1}=\left(mS_{\text{bar}}+nS_{\text{cross}}\right)CN_{\text{bar}},\\ CN_{2}=\left(mS_{\text{bar}}+nS_{\text{cross}}\right)CN_{\text{cross}}. \end{array}$$

**Figure 28:**
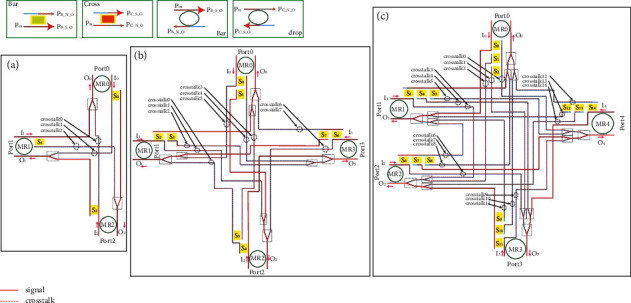
Analysis of router crosstalk model.

Here, *CN*_bar_ and *CN*_cross_ are the noise energy output by SPP switching at the bar and cross states, respectively, while for SPP MRR, it is the noise energy output at the bar and drop states. According to [31], the waveguide cross crosstalk is less than −50 dB. In the 5 × 5 router, the optical communication link maximum number of the waveguide cross is 9, so it is ignored in this paper.  Figure 27 shows the crosstalk model of the fault-tolerant optical router. Figures 28(a)–28(c)respectively establishes crosstalk models of 3 × 3, 4 × 4 and 5 × 5 optical routers.

**Figure 29:**
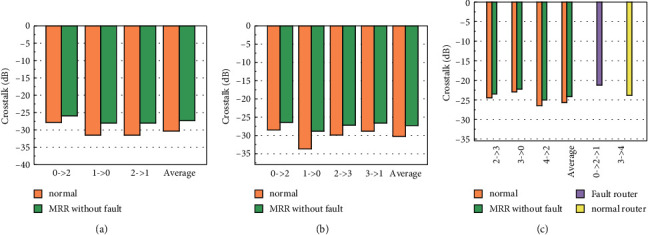
Simulation analysis of noise crosstalk.

Compared with the optical router without fault-tolerant MRR, the fault-tolerant optical router has one more SPP MRR at each input and output port. SPP MRR has two types of crosstalk: DROP and through. In fact, for the optical router, only noise crosstalk exists. Then, we experimentally simulated the noise crosstalk characteristic parameters of 3 × 3, 4 × 4 and 5 × 5 optical routers and plotted the experimental simulation results in Figure 29. Figures 29(a)–29(c) shows noise crosstalk characteristics of 3 × 3, 4 × 4, and 5 × 5 optical routers, respectively. According to the experimental simulation results, because each input and output port has one more SPP MRR, the noise crosstalk signal increases, and 3 × 3, 4 × 4, and 5 × 5 optical routers increase the average noise crosstalk by 2.95 dB, 2.96 dB, and 15.1 dB, respectively, which are 9.7%, 9.7%, and 5.8%, respectively. If an SPP switch or SPP MRR component of an optical router is faulty, other ports need to be reconfigured for fault tolerance. For 3 × 3 and 4 × 4 optical routers, if only one fault occurs, other ports will be occupied after the reconfigured optical link. The fault router mode does not need to be calculated when only internal noise crosstalk is considered. However, the noise crosstalk characteristics in fault router mode should be considered for a 5 × 5 optical router. In fault router mode, the reconfigured optical link is longer than the original optical link, and the noise of each optical device interferes with each other more. Therefore, the noise crosstalk degree is improved. Compared with MRR without fault mode, 2.95 dB noise crosstalk is increased.

The IL can be calculated using the following formula [22]:

(13) $$\begin{array}{c} IL_{T}=\sum IL_{\text{bar}}+\sum IL_{\text{cross}}+\sum IL_{\text{bend}}+\sum IL_{\text{crossing}}, \end{array}$$

where IL_bar_ and IL_cross_ are the IL in the bar state and in the cross state; according to [32], they are 0.4 dB and 2.1 dB, respectively. IL_bend_ and IL_crossing_ are the optical waveguide bends and the crossing loss; according to [21, 30], they are 0.005 dB and 0.04 dB, respectively.

**Figure 30:**
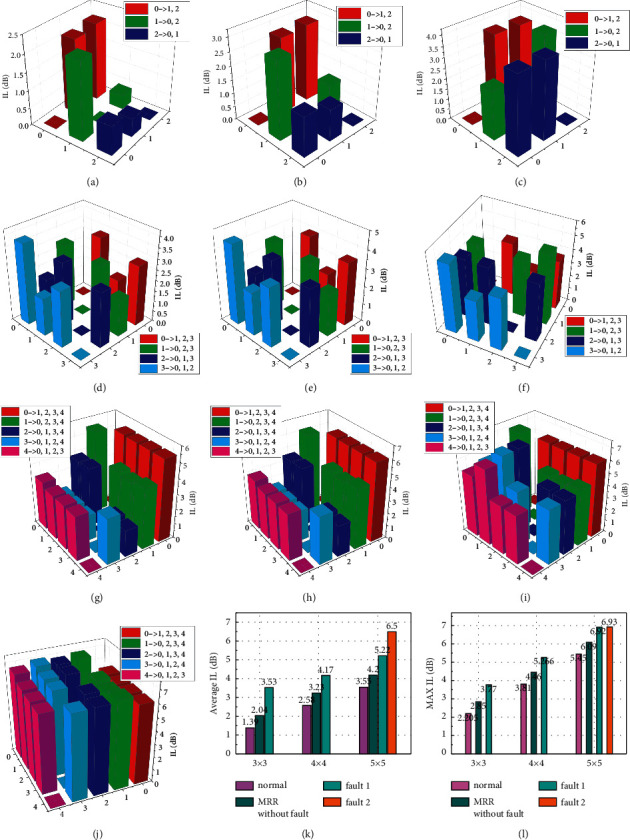
IL analysis: (a) IL of a 3 × 3 optical router in normal state. (b) IL of SPP MRR in a 3 × 3 optical router without failure. (c) IL of a 3 × 3 optical router, when the SPP MRR is faulty. (d) IL of a 4 × 4 optical router in normal state. (e) IL of SPP MRR in a 4 × 4 optical router without failure. (f) IL of a 4 × 4 optical router, when the SPP MRR is faulty. (g) IL of a 5 × 5 optical router in normal state. (h) IL of SPP MRR in 5 × 5 optical router without failure. (i) IL of the SPP MRR of the 5 × 5 optical router with one fault state. (j) IL of the SPP MRR in 5 × 5 optical router with two fault states. (k) Average IL for 3 × 3, 4 × 4, and 5 × 5 topologies. (l) Maximum IL for 3 × 3, 4 × 4, and 5 × 5 topologies.

Optical routers (3 × 3, 4 × 4 and 5 × 5) without MRR and with MRR and IL in the case of failure are simulated, and the results are plotted in Figure 30. Normal, MR without fault, fault 1 and fault 2 in subfigures (*k*) and (*l*) correspond to the IL of optical routers with no MRR and MRR at their input and output ports and with one or two failures, respectively. Figures 30(a)–30(j) shows the ILs of 3 × 3, 4 × 4, and 5 × 5 optical routers with no SPP MRR, with SPP MRR, and with SPP MRR failure, respectively. The fault-tolerant optical router has an extra SPP MRR in each input and output port, resulting in a larger IL of the communication link of the fault-tolerant router. According to the analysis of the average IL results in each case in Figure 30(k), for the 3 × 3 optical router, the average IL under MR without fault and with fault 1 is 0.65 dB and 2.14 dB higher than that under normal conditions, respectively. For 5 × 5 optical routers, the average IL is 0.65 dB, 1.67 dB, and 2.94 dB higher than normal for MR without fault, with fault 1, and with fault 2, respectively. When fault 2 is reconfigured, the longest optical communication link is found. As a result, the average IL of fault 2 increases the most (2.94 dB) compared with normal.

According to the data analysis of IL, when the full-dual-router is fault-tolerant, that is, when the port is added with fault-tolerant SPP MRR, the average IL of 3 × 3, 4 × 4 and 5 × 5 optical routers increases by 1 dB to 2 dB, while the maximum IL does not increase by 1 dB. In other words, when a fault-tolerant SPP MRR is added and a fault occurs, replanning the routing path has little influence on IL. On the other hand, the more ports an optical router has, the greater the IL is. Therefore, it is necessary to avoid excessive ports on an optical router when designing the network topology on an optical chip. The network topology on a 3D-mesh optical chip designed in this chapter avoids having more than five router ports in the center.

## 7. Conclusion

In this paper, an improved 3D mesh topology on an optical chip network is proposed first. The load balancing of the central router is balanced by improving the IP kernel mounting position of the central router. Then, 3 × 3, 4 × 4, and 5 × 5 optical routers with fault-tolerant performance are proposed and combined with a genetic optimization algorithm. The corresponding routing algorithm is proposed, and the overall reliability of the system is analyzed. The overall performance of topology design, fault-tolerant router, and routing algorithm in the system is analyzed by three parameters, namely, throughput rate, delay, and power consumption. Finally, the optical parameters of optical routing are analyzed. According to the data analysis results, the improved 3D-mesh structure designed for load balancing is effective and feasible. In terms of network data exchange, the router in the central position is reduced, the load is balanced, the port number of optical routers is reduced, and the IL is reduced. Finally, the optical parameters of the optical router are analyzed by simulation. The analysis results show that the IL and crosstalk noise of the optical router are less affected by setting the SPP MRR with a fault-tolerant function on the router port, and the IL and crosstalk noise are increased to 2.94 dB and 2.94 dB, respectively. The data analysis results also show that the more ports the optical router has, the greater the influence on IL and crosstalk noise parameters, which indicates that the improved 3D mesh topology is reasonable.

## Figures and Tables

**Algorithm 1:**
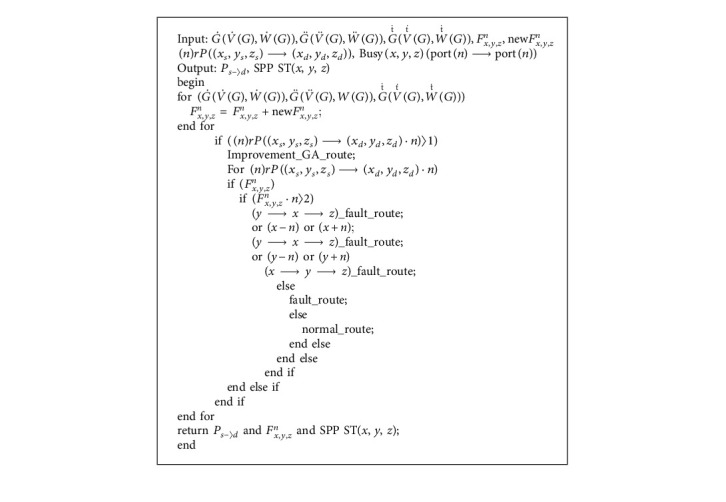
Fault-tolerant routing algorithm.

**Table 1:** Electrical layer simulation parameter setting.

Electronic control layer		
Clock frequency	1 GHz	

Packet size	32\64\128\256\512 bit	

Energy consumed	Dynamic_Electri_consumption_send	100 fJ/bit
	Dynamic_Electri_consumption_receive	100 fJ/bit
	Static_Electri_consumption_storage	2 fJ/bit
	Static_Electri_consumption_ctro	5 fJ/bit
	Dynamic_SPP_switch_consumption	13.1 fJ/bit
	Static_SPP_switch_consumption	3 fJ/bit

**Table 2:** Optical layer simulation parameter setting.

Optical layer	
OE/EO conversion delay	100 ps
Channel bandwidth bandwidth:	50 Gbps
Payload packet size Lpayload	32\64\128\256\512 bit
Modulator dynamic power Emod dyn	2 fJ/bit
Photodetector dynamic power consumption	50 fJ/bit
Photodetectors static power consumption	1 fJ/bit
Waveguide bend Lbend	0.005 dB

Crosstalk	SPP switch CNbar: 0.001933
	SPP switch CNcross: 0.00271
	SPP MRR_CNbar: 0.00876
	SPP MRR_CNcross: 0.06083

Insertion loss	SPP switch: BAR state 2.1 dB CROSS state 0.4 dB
	SPP MRR: BAR state 0.33 dB CROSS state 0.8 dB
	Bend: 0.005
	Crossing: 0.04

## Data Availability

The experimental data used to support the findings of this study are available from the corresponding author upon request.
